# Breakthrough Articles: Putting science first

**DOI:** 10.1093/nar/gku853

**Published:** 2014-10-07

**Authors:** David R. Corey, Jo Ann Wise, Keith R. Fox, Barry L. Stoddard

Over the past few years, there has been mounting concern that much of what is perceived as ‘cutting-edge’ research in the biological sciences cannot readily be reproduced or translated into new avenues for further development (1–3). One contributing factor to this problem may be that the scientific establishment disproportionately rewards investigators who publish their results in ‘prestige’ journals that have correspondingly high journal impact factors. Faced with enormous pressure to publish broadly visible and rapidly lauded research, investigators may feel they have no choice but to increase the scope of their work far beyond its actual capacity to yield reliable results, rather than striving to produce a less expansive (but fundamentally sound) study that would still drive important new ideas and experimental directions. The research community's over-reliance on journal impact factors, as a substitute for careful examination of the actual impact of a scientist's research, may well contribute to the accumulation of deficiencies in the research literature.

By favoring the authors of such papers in the competition for jobs, funding and promotions, the scientific community promotes a culture in which any research paper that is not published in one of these most desirable venues can be viewed as representing lower impact science. Due to this skewed system of values, papers published in high impact venues are sometimes judged on the perceived quality of the journal rather than their own individual merits. In response to such pressures, many scientists have been willing to jump through increasing numbers of hoops to publish in journals with exceptionally high impact factors.

The root of this problem does not lie solely with a handful of highly prestigious journals and their editors. Instead, the scientific community as a whole is to blame for fostering and acting on the assumption that a small group of publications hold a monopoly on the most outstanding of all science. The editors and staff of *Nucleic Acids Research*, motivated by the belief that *excellent science should be recognized and rewarded regardless of the journal in which it is published*, have chosen to highlight and actively promote ‘Breakthrough’ Articles as a regular feature of the journal. We hope that this new initiative to recognize and reward exceptional papers will further enhance the journal's standing among researchers.

A key goal in our decision to recognize ‘Breakthrough’ articles is to encourage investigators to publish their very best work in NAR. We are investing considerable effort into identifying and promoting studies that solve a long-standing problem in their field or provide exceptional new insight that will motivate and guide new research opportunities and directions. The scale of the research, as well the current attention being given to its corresponding field, is secondary to whether the study represents a true breakthrough in understanding.

Despite our appreciation of the dangers of relying too heavily upon journal impact factors as a substitute for systematic examination of the actual impact of a scientist's research or a paper's fundamental contribution to knowledge and understanding, we are nonetheless proud that NAR's own impact factor has risen substantially in recent years. We attribute the increasing number of citations per paper published in the journal, at least in part, to our long history of innovation in scientific publishing. In the 1970's, NAR was one of the first journals to speed the appearance of articles by having authors submit camera-ready manuscripts. In the 1990's, the journal centralized important research resources in its annual Database and Webserver issues, and in 2005 NAR and Oxford University Press joined the earliest vanguard of Open Access publishing by introducing completely free and unrestricted access to our content. We now continue this tradition of innovation with our commitment to identifying and actively nurturing Breakthrough science.

Instead of relying on editors to identify potentially high impact articles, we have instituted a “bottom-up” strategy in which our Breakthrough articles are usually first identified by peer reviewers. These are the scientists who are most likely to have the expertise to recognize a potential jewel in the form of a well defined and carefully conducted study and not be overly impressed by the presentation of a sweeping range of experiments. We have found that by carefully explaining our view on what constitutes truly exceptional research, our reviewers can accurately assess whether any study, regardless of size and scope, reports a true breakthrough within their field.

An author can also suggest that an article might be appropriate as a “Breakthrough” paper prior to or during the initial submission. This suggestion does not affect the level of scrutiny the paper receives, but can ensure that an editorial board member is consulted early in the process and thereby allows a decision to be made more quickly.

Once a potential Breakthrough paper has been identified during peer review, we solicit a second level of review from members of our editorial board and outside experts. After these rounds of peer evaluation, senior and executive editors of NAR examine the manuscript before coming to a final decision. Manuscripts that pass this editorial scrutiny are then edited and suggestions are made to improve their accessibility to a broad audience. NAR prides itself on rapid deliberations, and the average time from submission to first decision is 21 days. The time required for the multiple rounds of evaluation for Breakthrough articles is seldom more than a few days.

The broad research expertise within the NAR family of editors and editorial board members (all of whom are practicing scientists) helps ensure that publication of outstanding research will not be blocked or delayed as the result of bias. Another significant advantage is that NAR has no page limits, allowing full disclosure of important data within the main text and ample space for thoughtful discussion of results. Finally, by accepting that a breakthrough can often be found within a focused but technically outstanding study, we resist the temptation to demand additional experiments and broadened scope beyond what is necessary or advisable.

Beyond the initial designation of a study as a ‘Breakthrough’, we further promote the outstanding science in these studies through ‘NAR Breakthrough Paper Alerts’. These documents, which are circulated to the organizers and writers of numerous online and printed research abstract services and topical science magazines, describe the exact manner in which the study represents a breakthrough, as well as the contribution that the authors, their institution and their funding agencies have made to the work.

In a little over a year NAR has already published nineteen Breakthrough articles. We hope that our decision to recognize and reward exceptional papers will prove useful for a broad audience, ranging from research experts in the specific discipline, to students looking for a compelling paper that will withstand the scrutiny of the harshest journal club. While our initiative will not single-handedly solve the problem of identifying high quality scholarship independent of the journal in which it is published, we hope it will be one of many productive responses from the scientific community that help bring the focus back towards science with real and lasting value.

**Table:** 

David R. Corey	Keith R. Fox
Jo Ann Wise	Barry L. Stoddard
*Executive Editors*	*Senior Executive Editors*
